# Hypoxia-inducible factor-1α–mediated metabolic reprogramming in sepsis-associated organ dysfunction: organ-specific metabolic mismatch and therapeutic perspectives

**DOI:** 10.3389/fimmu.2026.1998563

**Published:** 2026-10-12

**Authors:** Yuge Wu, Jiantao Lu, Yue Mo, Yaoqi Xu, Xiaoxi Yan, Caifen Yan, Yingzhen Wang

**Affiliations:** 1 Emergency Center, The Second Hospital and Clinical Medical School, Lanzhou University, Lanzhou, Gansu, China; 2 Department of Critical Care Medicine, The Second Hospital and Clinical Medical School, Lanzhou University, Lanzhou, Gansu, China

**Keywords:** hypoxia-inducible factor-1α, metabolic flexibility, metabolic reprogramming, mitochondrial dysfunction, organ-specific metabolic mismatch, sepsis

## Abstract

Sepsis-associated organ dysfunction arises from immune dysregulation, heterogeneous microcirculatory perfusion, impaired oxygen utilization, and cellular metabolic disturbances. Hypoxia-inducible factor-1α (HIF-1α) links oxygen sensing and inflammatory signaling to substrate use and mitochondrial function, but its effects vary with cell type, organ energy demand, and disease stage. This review uses metabolic flexibility and organ-specific metabolic mismatch to integrate these differences. Metabolic flexibility denotes the capacity to adjust energy-producing pathways during stress and restore efficient oxidative metabolism after stress subsides; mismatch develops when cellular energy production no longer meets immediate organ function or repair requirements. HIF-1α-related glycolysis supports early antimicrobial responses in myeloid cells and short-term hypoxic tolerance in some parenchymal cells. Persistent glycolysis combined with restricted pyruvate and fatty acid oxidation and impaired mitochondrial renewal may instead prolong inflammation and limit barrier repair, tubular transport, myocardial contraction, hepatic clearance, and neurological recovery. Differences among the lungs, kidneys, heart, intestine, liver, and brain indicate that global HIF activation or inhibition is unlikely to produce uniform benefit. Cell type-resolved localization, dynamic metabolic flux, mitochondrial function, and organ-level endpoints are therefore required to distinguish adaptive reprogramming from persistent bioenergetic dysfunction and to guide phase-matched metabolic intervention.

## 1 Introduction

Sepsis is life-threatening organ dysfunction caused by a dysregulated host response to infection and remains a major cause of mortality and long-term disability worldwide (1, 2). Pathogen- and damage-associated molecular patterns activate inflammatory, immune, coagulation, endothelial, microcirculatory, and neuroendocrine networks. Pro-inflammatory and immunosuppressive responses may coexist, with their relative contributions varying by cell type, infection site, affected organ, and time (3, 4). This heterogeneity helps explain why improvement in circulating inflammatory markers does not necessarily parallel organ recovery and why single inflammatory targets have rarely produced consistent clinical benefit.

Organ dysfunction is determined not only by oxygen delivery but also by microvascular distribution, cellular oxygen extraction, substrate utilization, and mitochondrial competence. Even after fluid resuscitation and vasoactive therapy restore macrohemodynamic variables, regional hypoperfusion and impaired oxygen use may persist. Cytopathic hypoxia refers to inadequate cellular oxygen utilization despite available oxygen and substrates, most commonly because nitric oxide, reactive oxygen species, redox imbalance, loss of mitochondrial membrane potential, or respiratory-chain injury restricts oxidative phosphorylation (5, 6). It is therefore distinct from delivery-limited hypoxia, although both may coexist within the same organ. Blood pressure, oxygenation, and lactate therefore cannot independently define the metabolic state of a specific organ, and functional depression does not necessarily indicate widespread cell death.

Under normoxia, HIF-1α is hydroxylated by prolyl hydroxylase domain proteins, recognized by von Hippel-Lindau protein, and degraded through the ubiquitin-proteasome pathway. Hypoxia or impaired hydroxylation stabilizes HIF-1α, allowing heterodimerization with HIF-1β and transcription of hypoxia-responsive genes (7). During sepsis, Toll-like receptor/nuclear factor-κB and phosphoinositide 3-kinase/AKT/mechanistic target of rapamycin signaling, reactive oxygen and nitrogen species, and tricarboxylic acid (TCA) cycle intermediates can activate HIF-1α independently of marked hypoxemia. Succinate and pyruvate kinase M2 (PKM2) further reinforce HIF-1α-dependent glycolytic and inflammatory programs (8, 9). HIF-1α increases glucose transport, glycolysis, lactate dehydrogenase A, and pyruvate dehydrogenase kinase, thereby limiting pyruvate oxidation and influencing fatty acid oxidation, redox balance, and mitochondrial quality control (7).

The consequences are cell-specific. Baseline substrate preference is cell- and region-specific rather than uniform within an organ. Glycolysis rapidly supports antimicrobial activity in monocytes, macrophages, and neutrophils, whereas lymphocyte activation and exhaustion depend on coordinated glucose, glutamine, and lipid metabolism. Later, some circulating immune cells develop concurrent suppression of glycolysis and oxidative phosphorylation, reduced cytokine responsiveness, and impaired pathogen clearance, consistent with immunometabolic paralysis (10, 11). Parenchymal requirements differ: renal proximal tubules and cardiomyocytes depend strongly on mitochondrial oxidation for active transport and contraction; alveolar, intestinal, and vascular barrier cells require energy for transport, junctional integrity, and regeneration; hepatocytes must sustain biosynthesis and clearance; and neurons require continuous oxidative energy for synaptic activity. Thus, the same HIF signal can support short-term survival in one cell population while worsening inflammation or delaying functional recovery in another.

This distinction is clinically important because hypoxemia, oliguria, delayed lactate clearance, myocardial depression, impaired hepatic clearance, and altered consciousness may persist even when systemic perfusion appears adequate. Each phenotype can arise from a different combination of microvascular maldistribution, reversible functional suppression, altered substrate selection, and structural mitochondrial injury. Moreover, inflammatory resolution does not necessarily restore oxidative metabolism: mitochondrial biogenesis, cofactor regeneration, substrate oxidation, and cellular redifferentiation may recover on different timescales (12–14). A useful framework must therefore connect molecular signaling not only to survival or injury markers but also to the energy-consuming function that defines each organ.

Previous reviews have emphasized HIF signaling, immunometabolism, or therapeutic targets (15–17), but have less often integrated cell identity, organ energy demand, disease phase, and functional outcome. We therefore use two linked concepts. Metabolic flexibility is the ability to change fuel pathways during stress and restore efficient oxidation afterward. Organ-specific metabolic mismatch is operationally defined here by the coexistence of three features: substrate utilization that is discordant with cellular workload, inadequate ATP production or respiratory reserve relative to oxygen and substrate availability, and persistent failure of the relevant organ function or repair program. HIF-1α expression, mitochondrial dysfunction, or transient adaptive hypometabolism alone is insufficient to establish metabolic mismatch. No validated clinical threshold currently separates these states; therefore, metabolic mismatch should be evaluated as a longitudinal composite rather than inferred from HIF-1α expression or circulating lactate alone. Across six organs, we assess whether HIF-related metabolic changes improve gas exchange, active transport, contraction, barrier function, metabolic clearance, or neurological recovery and consider the implications for cell-selective and phase-matched treatment.

## 2 Literature search and evidence classification

This narrative review was informed by targeted PubMed/MEDLINE searches through September 2026. Search combinations paired sepsis, septic shock, endotoxemia, or lipopolysaccharide with HIF-1α, hypoxia, cytopathic hypoxia, glycolysis, PDH/PDK, fatty acid oxidation, ketone metabolism, mitophagy, ferroptosis, mitochondrial dysfunction, metabolomics, and organ- or cell-specific terms. Reference lists of relevant reviews and primary studies were screened for additional reports. Priority was given to human sepsis studies, followed by clinically relevant animal sepsis models, endotoxemia or LPS-based studies, *in vitro* studies, and non-sepsis mechanistic models when sepsis-specific evidence was unavailable. No formal meta-analysis or risk-of-bias assessment was performed.

Evidence is identified throughout the review as human sepsis, animal sepsis, endotoxemia or LPS-based evidence, *in vitro* evidence, or non-sepsis mechanistic evidence. Human associations are not treated as proof of organ-specific metabolic flux, and mechanisms extrapolated from non-sepsis models are labeled as indirect. This hierarchy also distinguishes direct HIF-1α effects from intersecting and parallel metabolic pathways.

## 3 Major processes of HIF-1α-mediated metabolic reprogramming

### 3.1 Combined effects of inflammation and hypoxia on glycolysis and mitochondrial metabolism

Endothelial glycocalyx loss, altered vascular reactivity, leukocyte adhesion, microthrombi, and edema reduce functional capillary density and create spatially heterogeneous oxygen delivery. Hypoperfused microvascular regions may persist despite normalization of blood pressure and cardiac output, while inflammatory oxygen consumption and increased epithelial repair demand further widen cell-level differences in oxygen exposure (18, 19). In parallel, lipopolysaccharide-induced Toll-like receptor 4/nuclear factor-κB signaling increases HIF-1α transcription, and PI3K/AKT/mTOR signaling promotes protein synthesis. Tumor necrosis factor-α, interleukin-1β, and reactive oxygen species also alter prolyl hydroxylase activity, allowing HIF-1α signaling before tissue oxygen tension falls substantially (7, 17). Early activation supports myeloid antimicrobial activity, whereas persistent stimulation can maintain innate immune glycolysis while adaptive immune responses decline (10, 11).

Inflammatory and hypoxic signals redirect glucose-derived carbon from mitochondrial oxidation toward lactate. Inducible nitric oxide synthase-derived nitric oxide inhibits cytochrome c oxidase, while reactive oxygen species and peroxynitrite damage respiratory complexes, mitochondrial membranes, and mitochondrial DNA (5, 13). Electron leakage then amplifies mitochondrial reactive oxygen species, and released mitochondrial components perpetuate inflammation as damage-associated molecular patterns. HIF-1α induces glucose transporter 1, hexokinase 2, PFKFB3, PKM2, lactate dehydrogenase A, and pyruvate dehydrogenase kinase 1. Pyruvate dehydrogenase kinase inhibits pyruvate dehydrogenase and limits pyruvate entry into the TCA cycle (7–9). Glycolytic rates and lactate production may therefore increase or remain relatively preserved while downstream glucose oxidation falls; PDH inhibition uncouples glycolysis from mitochondrial glucose oxidation. This shift may temporarily reduce demand on an injured respiratory chain, but prolonged HIF stabilization and glycolysis become maladaptive if pyruvate oxidation, fatty acid oxidation, and mitochondrial biogenesis fail to recover.

Glycolysis also supports redox defense and biosynthesis. The pentose phosphate pathway generates NADPH for glutathione regeneration and lipid synthesis, while glycolytic intermediates supply nucleotide, amino acid, and glycosylation pathways (20). Nuclear PKM2 cooperates with HIF-1α to promote inflammatory transcription, including interleukin-1β (9). Lactate can be exchanged through monocarboxylate transporters and oxidized by tissues with preserved mitochondrial capacity, but excessive accumulation and acidification alter endothelial and immune function (21). Because blood lactate also reflects adrenergic stimulation and hepatic and renal clearance, it cannot be equated with systemic tissue hypoxia (22).

TCA intermediates act as signaling molecules. In inflammatory macrophages, citrate export supports lipid and mediator synthesis, whereas succinate inhibits prolyl hydroxylases, stabilizes HIF-1α, and promotes interleukin-1β. Succinate oxidation through succinate dehydrogenase also generates mitochondrial reactive oxygen species (8, 23). Itaconate, derived from cis-aconitate, can inhibit succinate dehydrogenase and constrain inflammation (24); the cell-permeable derivative 4-octyl itaconate can also activate NRF2 by modifying Kelch-like ECH-associated protein 1 (25). Because itaconate derivatives differ from endogenous itaconate in electrophilicity and cellular disposition, their effects require model-specific interpretation. Overall, early glycolysis can be adaptive, but persistence after pathogen control signals failure to restore efficient oxidation.

The relationship between HIF-1α and mitophagy is conditional rather than universal. HIF-1α can transcriptionally induce BNIP3- or BNIP3L-mediated receptor mitophagy, but sepsis can also activate mitochondrial quality-control pathways through membrane depolarization, oxidative stress, calcium overload, and inflammatory injury independently of HIF. Controlled mitophagy removes damaged mitochondria and limits reactive oxygen species and mitochondrial damage-associated molecular pattern release, whereas persistent clearance without adequate mitochondrial biogenesis can reduce respiratory reserve. Its net effect therefore depends on the initiating signal, target cell, organ workload, and disease phase (7, 13).

The temporal transition is gradual rather than a fixed three-stage sequence. During early infection, reduced mitochondrial oxygen consumption and selective mitophagy may limit oxidative stress while glycolysis supplies rapid ATP and biosynthetic intermediates. If pathogen burden, inflammatory signaling, and mitochondrial injury continue, impaired NADH reoxidation and restricted pyruvate entry can reinforce lactate production and redox imbalance (13). During recovery, restoration of pyruvate dehydrogenase activity, fatty acid oxidation, respiratory reserve, and mitochondrial biogenesis determines whether the initial response remains adaptive (13, 14). These processes overlap among cells and organs; consequently, timing must be defined by biological state and functional recovery rather than elapsed time alone.

To improve interpretive clarity, this review distinguishes evidence that directly supports HIF-1α-dependent regulation from evidence indicating association or pathway crosstalk. Stronger support for a direct HIF-1α effect is provided by cell-specific HIF manipulation, regulation through a hypoxia-response element, or target engagement accompanied by changes in metabolic flux or organ function. GLUT1, LDHA, PDK1, and BNIP3/BNIP3L have been reported as HIF-responsive targets in multiple experimental contexts. By contrast, NF-κB, mTOR, succinate, reactive oxygen species, and nuclear PKM2 often regulate or cooperate with HIF-1α, whereas PGC-1α, NRF2/HO-1, ferroptosis, C1q, and GPR43 may represent intersecting, parallel, or indirect mechanisms. Their precise relationships to HIF-1α should therefore be interpreted according to the cell type, disease stage, and experimental model.

### 3.2 Fatty acid and amino acid metabolism, redox homeostasis, and persistent metabolic effects

Septic metabolism cannot be reduced to glycolysis. Myocardial PPARα-related fatty acid oxidation may rise transiently during early polymicrobial sepsis (26), whereas hepatic PPARα and lipid oxidation are suppressed (27). PPARα deficiency also worsens fatty acid utilization and proximal tubular injury in experimental sepsis-associated acute kidney injury (28). These results show that fatty acid oxidation varies by organ, genetic background, and observation time. Amino acid profiles are likewise shaped by systemic catabolism, nutrition, and organ clearance. Glutamine, arginine, tryptophan, and branched-chain amino acids participate in anaplerotic, redox, and immune pathways, but their organ- and cell-specific fluxes in sepsis remain incompletely resolved. Clinical metabolite concentrations therefore do not directly represent cell-specific flux or prove that supplementing a single substrate will improve bioenergetics (20).

The Randle cycle links fatty acid oxidation to suppression of PDH-mediated glucose oxidation, whereas glucose and insulin restrain fatty acid use. In sepsis, however, concurrent impairment of PDH, fatty acid oxidation, and mitochondrial respiration may limit this reciprocal compensation. Reduced glucose oxidation therefore does not necessarily increase fatty acid or ketone oxidation, and substrate availability may not translate into effective ATP production (7, 13, 26–28).

Lipotoxicity develops when fatty acid uptake exceeds mitochondrial β-oxidation, safe esterification, or export. Long-chain acyl-coenzyme A, acylcarnitines, diacylglycerols, ceramides, and oxidizable phospholipids can then impair membranes, insulin signaling, calcium handling, and mitochondrial respiration and increase reactive oxygen species. Neutral triglyceride droplets may buffer excess fatty acids and should not be equated automatically with toxicity. Lipid accumulation in septic kidney, heart, or liver is therefore most informative when paired with fatty acid oxidation flux and measurements of specific lipid intermediates (27, 28).

Substrate oxidation depends on redox cofactors. NAD+/NADH transfers electrons among glycolysis, the TCA cycle, fatty acid oxidation, and the respiratory chain, whereas NADPH regenerates glutathione and thioredoxin. Respiratory-chain injury restricts NADH reoxidation, and poly(ADP-ribose) polymerase activation after oxidative DNA damage accelerates NAD+ depletion. Experimental kidney injury links PGC-1α-dependent NAD+ biosynthesis to tubular oxidative recovery (29), whereas monocyte models show phase-dependent coordination of SIRT1, RELB, and SIRT3 during inflammatory adaptation (30). These observations support redox-sensitive metabolic recovery but remain cell- and context-dependent.

The consequences of altered substrate use also depend on basal metabolic specialization. Baseline substrate preference is relative and cell-specific. Pulmonary endothelial cells are strongly glycolytic, whereas alveolar epithelial cells retain mixed use of fatty acids, pyruvate, and amino acids. Cortical proximal tubules and cardiomyocytes depend heavily on fatty acid oxidation and oxidative phosphorylation; medullary nephron segments operate at lower oxygen tension with segment-dependent oxidative and glycolytic capacity. Hepatocyte fuel use is zonated, colonocytes preferentially oxidize butyrate, and astrocytes have greater glycolytic and glycogen capacity than neurons, which rely mainly on oxidative metabolism. These preferences are not exclusive and change with oxygen availability, nutrition, hormonal signaling, and injury. Myeloid and some barrier cells can maintain selected functions through glycolysis for a limited period, whereas cardiomyocytes and proximal tubular cells require high-capacity oxidative phosphorylation to sustain contraction or active transport. Circulating amino acid and lipid abnormalities mainly describe the systemic substrate environment and may be driven by muscle proteolysis, feeding, hormonal stress, or impaired hepatic and renal clearance. They cannot determine whether a given cell is taking up, oxidizing, storing, or exporting a substrate. This distinction limits direct inference from clinical metabolomics to organ-specific supplementation strategies.

Metabolic effects may outlast the initiating stimulus without constituting a uniform HIF-driven metabolic memory in sepsis. Remodeling of glycolysis and the TCA cycle alters acetyl-CoA, α-ketoglutarate, succinate, fumarate, lactate, and NAD+, which can influence chromatin regulation. Trained-immunity models implicate glutaminolysis, fumarate accumulation, and associated histone modifications in sustained transcriptional responses (31); histone lactylation provides a separate mechanism by which accumulated lactate may influence gene expression (32). Neither mechanism has been established as a uniform HIF-dependent program in sepsis. Conversely, sepsis-associated immunoparalysis is characterized by low glycolytic and oxidative responsiveness (10). Longitudinal assessment of HIF activity, metabolic flux, chromatin state, and organ recovery in defined cell subsets is therefore needed to distinguish transient adaptation, trained immunity, and immunoparalysis. The integration of hypoxic, inflammatory, and metabolic signals by HIF-1α, together with the context-dependent transition from metabolic flexibility to organ-specific metabolic mismatch, is illustrated in **Figure 1**.

**Figure 1:**
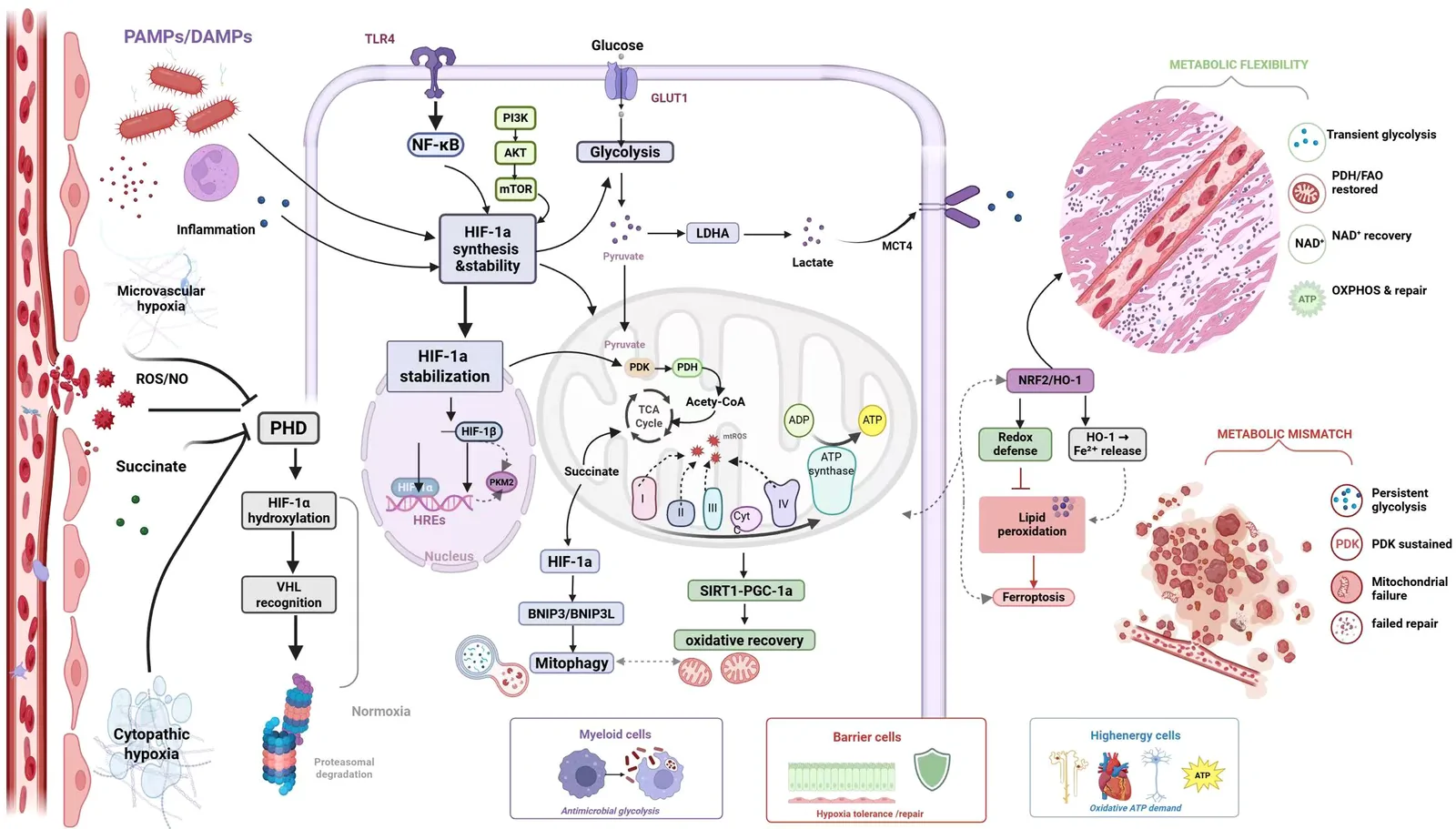
HIF-1α-driven metabolic reprogramming in sepsis: from stress sensing to metabolic flexibility or organ-specific metabolic mismatch. HIF-1α-driven metabolic reprogramming in sepsis. Hypoxia, inflammatory signals, ROS/NO, and succinate stabilize HIF-1α, promoting glycolysis and restricting pyruvate oxidation. Depending on cell type, mitochondrial reserve, and disease phase, this response may support transient adaptation and repair or progress to persistent inflammation, mitochondrial dysfunction, and organ-specific metabolic mismatch. NRF2/HO-1 and ferroptosis are presented as intersecting rather than universal downstream pathways.

## 4 Organ-specific metabolic reprogramming and functional outcomes

### 4.1 Inflammatory glycolysis and barrier metabolic imbalance in the lung

Sepsis-associated lung injury reflects disruption of the alveolar-capillary barrier, heterogeneous perfusion, edema, microthrombosis, and ventilation-perfusion mismatch (18, 33). Alveolar exposure to air does not ensure adequate oxygenation of epithelial, endothelial, or infiltrating immune cells because local ventilation, perfusion, diffusion, and mitochondrial oxygen utilization all contribute. Their metabolic demands also differ: myeloid cells require rapid energy for antimicrobial activity, endothelial cells maintain vascular integrity, and alveolar type II cells sustain ion transport, surfactant synthesis, and epithelial repair.

Alveolar macrophages and neutrophils are major sources of inflammatory glycolysis. Succinate–HIF-1α signaling supports inflammatory transcription in macrophages (8), whereas HIF-1α-dependent glycolysis promotes the survival of hypoxic tissue-resident alveolar macrophages but may prolong inflammation (34). HIF-1α/NF-κB signaling also extends neutrophil survival under hypoxic conditions and supports migration and degranulation (35). These responses can promote early pathogen control, yet persistent neutrophils release proteases, reactive oxygen species, and extracellular traps that injure the epithelial–endothelial barrier.

Pulmonary endothelial cells normally rely heavily on glycolysis (36). Single-cell studies have identified altered metabolic programs in capillary endothelial subsets during sepsis-associated lung injury (37), including SLC2A1-related glucose uptake and glycolytic reprogramming (38). Transcript abundance, however, does not establish flux. Endothelial HIF-1α supports FOXM1-dependent vascular repair during the resolution of inflammatory lung injury (39). Persistent endothelial glycolytic activation may nevertheless contribute to barrier dysfunction under sustained inflammatory conditions, although direct causal evidence in sepsis remains limited. Alveolar type II cells likewise use HIF-related glycolysis for short-term survival and repair (40). A 2024 study found that type II cell-specific loss of Cpt1a or Acadl reduced long-chain fatty acid oxidation but preserved ATP through pyruvate and glutamine oxidation, reduced CXCL2 and neutrophilic inflammation, and altered surfactant phospholipids (41). This illustrates that reduced fatty acid oxidation does not necessarily equal energy failure when alternative substrates remain available.

Mitochondrial quality control provides an additional link between acute survival and later repair. In the lung, infection, inflammatory oxidants, and mitochondrial depolarization may initiate mitophagy, while HIF-1α can contribute through BNIP3/BNIP3L signaling (7, 13). In experimental bacterial pneumonia, alveolar type II cells activate mitophagy and mitochondrial biogenesis in parallel, suggesting that clearance and replacement need to remain coordinated (42). Limited removal of damaged mitochondria may reduce reactive oxygen species and mitochondrial damage signals, whereas persistent clearance without adequate biogenesis may lower respiratory capacity. LC3, BNIP3, or PGC-1α expression alone is therefore insufficient to define recovery; autophagic flux, mitochondrial abundance and respiration, epithelial transport, surfactant composition, and endothelial permeability provide more direct functional context.

Pulmonary mismatch therefore reflects persistent inflammatory glycolysis in myeloid cells alongside the mitochondrial, lipid, and differentiation requirements of barrier repair. Studies should resolve target cells and treatment timing and assess pathogen burden, vascular permeability, alveolar fluid clearance, oxygenation, lung compliance, and cellular repair rather than tissue HIF-1α alone. The organ-specific metabolic responses of the lung, kidney, heart, intestine, liver, and brain, as well as their coupling through circulating substrates, metabolites, and inflammatory mediators, are summarized in **Figure 2**.

**Figure 2:**
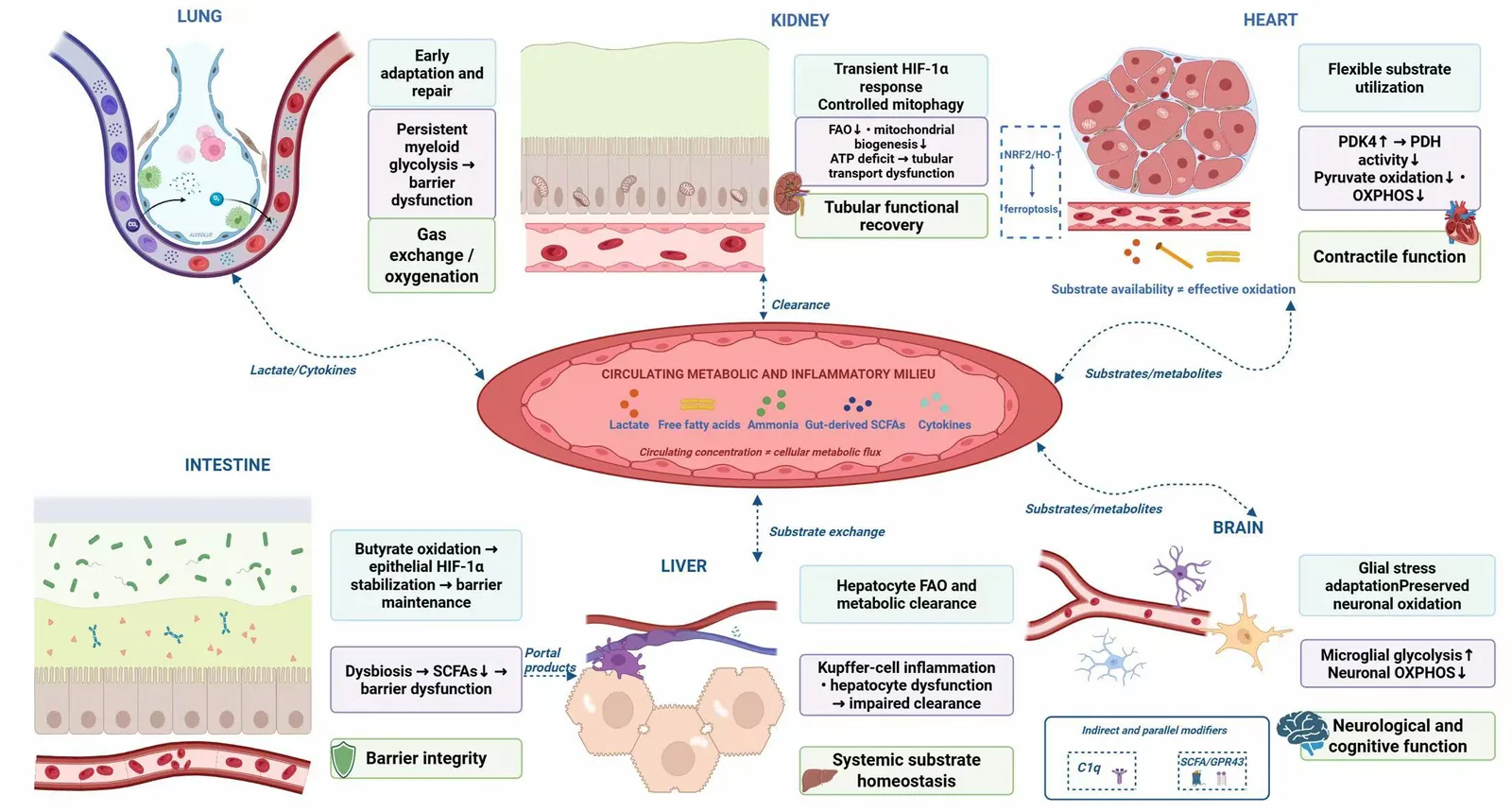
Organ-specific metabolic reprogramming and interorgan metabolic coupling in sepsis. Organ-specific metabolic reprogramming and interorgan coupling in sepsis. Metabolic responses differ among the lung, kidney, heart, intestine, liver, and brain according to cellular energy demand and mitochondrial dependence. These organs are interconnected through circulating substrates, metabolites, and inflammatory mediators. Organ recovery depends on the balance between adaptive HIF signaling, oxidative metabolism, substrate utilization, and metabolite clearance.

### 4.2 Metabolic adaptation and impaired repair in energy-demanding renal tubules

Sepsis-associated acute kidney injury combines heterogeneous intrarenal perfusion, endothelial dysfunction, inflammation, mitochondrial injury, and tubular metabolic suppression (43, 44). Proximal tubules continuously consume ATP for sodium reabsorption, solute transport, and polarity and depend predominantly on mitochondrial oxidative phosphorylation and fatty acid oxidation. Local oxygenation may remain impaired despite preserved or restored total renal blood flow.

The kidney’s corticomedullary oxygen gradient complicates metabolic interpretation. Cortical proximal tubules have high ATP demand, limited glycolytic reserve, and depend largely on fatty acid oxidation and oxidative phosphorylation. The outer medullary thick ascending limb also requires oxidative ATP for sodium transport but operates at lower oxygen tension, whereas more distal and inner medullary segments have greater glycolytic capacity (43, 44). Sepsis may therefore produce segment-specific hypoxia and impaired oxygen utilization even when total renal blood flow is preserved. Temporary reductions in transport and differentiation may conserve ATP, so oliguria or altered filtration can occur without extensive necrosis; conversely, recovery of creatinine or urine output does not confirm restoration of substrate oxidation.

Hypoxia, reactive oxygen species, and inflammation jointly regulate tubular HIF-1α. In experimental acute kidney injury, macrophage-associated nuclear factor-κB signaling increases tubular HIF-1α transcription and is associated with protection (45). HIF-1α-related glycolysis may provide limited energy when oxidative phosphorylation is impaired, but the low basal glycolytic capacity of proximal tubules cannot sustain transport and repair. Sepsis and other kidney injuries also suppress PPARα-, PGC-1α-, and carnitine-dependent fatty acid oxidation (28, 46). When fatty acid uptake exceeds oxidation or safe storage, acylcarnitines, diacylglycerols, and ceramides may accumulate and disturb membranes, redox balance, and signaling, providing a potential basis for tubular lipotoxicity. Because part of this evidence comes from chronic kidney injury, its relevance to early sepsis should be interpreted cautiously.

Mitochondrial and redox pathways influence reversibility. Tubular HIF-1α/BNIP3-mediated mitophagy reduces oxidative stress and apoptosis in ischemia-reperfusion injury, although direct septic evidence is limited (47); mitochondrial depolarization and oxidant injury may also activate mitophagy without requiring HIF-1α. NRF2/HO-1 is an intersecting redox network rather than an exclusive HIF downstream pathway. Experimental sepsis-associated kidney injury links NRF2 activation to improved mitochondrial homeostasis (48), whereas HO-1 may limit heme-associated oxidative injury but can also increase labile iron when storage and export are insufficient (49). Ferroptosis-related changes in GPX4, ACSL4, iron, and lipid peroxidation have been reported in cecal ligation and puncture and lipopolysaccharide models (50, 51). The renal consequences principally involve tubular transport and repair; comparable cell-resolved evidence in other septic organs remains less developed. Ferroptosis is therefore treated as a parallel redox injury process that may intersect with HIF signaling rather than as a uniform HIF downstream mechanism.

Recovery requires renewed fatty acid oxidation, NAD+ synthesis, mitochondrial biogenesis, and redifferentiation. In endotoxemic mice, renal PGC-1α expression and oxidative phosphorylation-related genes decline early and recover in parallel with renal function; PGC-1α deficiency delays both mitochondrial and renal recovery (52). PGC-1α-dependent NAD+ biosynthesis further supports restoration of tubular oxidative metabolism (29). Tubular mismatch is therefore the coexistence of high transport and repair demands with persistently inefficient energy production. Evaluation should combine cell-resolved HIF-1α, NRF2/HO-1, ferroptosis, and mitophagy with tubular oxygen consumption, fatty acid oxidation flux, urinary injury markers, urine output, creatinine, and long-term function.

### 4.3 Impaired cardiac substrate switching and insufficient energy for contraction

Sepsis-associated myocardial dysfunction may involve left- or right-ventricular systolic dysfunction, impaired relaxation, and reduced strain, but ejection fraction is strongly influenced by preload, afterload, vasoactive drugs, and mechanical ventilation (53). Healthy myocardium flexibly oxidizes fatty acids, glucose, lactate, ketone bodies, and amino acids, with fatty acids providing much of its ATP (54). During sepsis, circulating substrates may rise through catecholamine stimulation and catabolism, while nitric oxide, reactive oxygen species, and inflammation inhibit the respiratory chain, mitochondrial membrane potential, and pyruvate oxidation. Myocardial mitochondrial proteomics after cecal ligation and puncture identified altered pyruvate and fatty acid metabolism, increased pyruvate dehydrogenase kinase 4, reduced pyruvate dehydrogenase activity, and impaired pyruvate-driven respiration (55).

HIF-1α increases myocardial glycolysis while restricting mitochondrial pyruvate use. Systemic 2-deoxyglucose improved inflammation, apoptosis, cardiac function, and survival in a cecal ligation and puncture model, but could not establish a cardiomyocyte-specific mechanism (56). More direct evidence showed that lipopolysaccharide-induced excess cardiomyocyte HIF-1α inhibited respiration through inducible nitric oxide synthase and nitric oxide; partial myocardial HIF-1α deficiency attenuated mitochondrial and contractile dysfunction (57). Transient HIF activation may support acute hypoxic tolerance, but sustained activation cannot replace the high oxidative demand of adult myocardium.

Fatty acid oxidation varies with model and disease phase. It can rise transiently during early polymicrobial sepsis, and PPARα deficiency impairs this response (26). By contrast, reduced myocardial PPARα expression and fatty acid oxidation have been reported in other septic models and are associated with impaired KLF5-dependent regulation of PPARα (58). When fatty acid uptake exceeds mitochondrial oxidation, acylcarnitines, diacylglycerols, and ceramides may accumulate and interfere with membrane signaling and mitochondrial function, providing a potential basis for myocardial lipotoxicity. Changes in fatty acid oxidation should therefore be interpreted together with ATP production, lipid intermediates, and mechanical function. Lactate and ketone bodies can support the heart only when mitochondrial oxidation remains available.

Bioenergetic failure and calcium dysregulation can amplify one another. Reduced ATP limits sarcoplasmic reticulum calcium reuptake, sodium-potassium pump activity, relaxation, and myofilament cycling; cytosolic and mitochondrial calcium overload can then lower membrane potential, increase reactive oxygen species, and further restrict oxidative phosphorylation (53, 59). Mitophagy may remove severely depolarized mitochondria, but sustained clearance without replacement can reduce respiratory reserve. Its dominant triggers may differ among organs: bacterial and inflammatory stress is prominent in lung epithelium, HIF-1α/BNIP3 evidence in renal tubules derives mainly from ischemia-reperfusion, and calcium- and oxidant-related depolarization may be particularly relevant in cardiomyocytes (42, 47, 53, 59). During recovery, coordinated restoration of NAD+ homeostasis, mitochondrial biogenesis, pyruvate oxidation, and fatty acid use is more informative than a single autophagy or biogenesis marker. PDK4 silencing or inhibition improves pyruvate metabolism, mitochondrial structure, and contraction in experimental sepsis (55, 60). Cardiac mismatch is thus characterized by available circulating substrates but inadequate oxidative ATP for mechanical work.

### 4.4 Gut microbiota-derived metabolites and HIF-1α-related barrier adaptation

Mesenteric hypoperfusion, edema, inflammation, and mitochondrial injury disturb epithelial oxygen balance during sepsis. Cell death, tight-junction remodeling, and mucus loss increase permeability and microbial translocation, potentially affecting the liver through the portal vein and distant organs through mesenteric lymph and systemic circulation (61). Intestinal dysfunction also includes impaired absorption, epithelial renewal, mucosal immunity, and microbiota composition.

Intestinal epithelial oxygenation and luminal oxygenation refer to related but distinct compartments. Epithelial oxygenation describes oxygen available within the mucosa and epithelial cells from the local microcirculation, whereas luminal oxygenation refers to oxygen reaching the mucosal surface and gut lumen. Colonocyte oxidation of microbiota-derived butyrate consumes oxygen, maintains physiological epithelial hypoxia, and limits oxygen diffusion into the lumen, thereby supporting HIF-1α-dependent barrier programs (62–64). Fasting, interrupted enteral nutrition, antibiotics, or loss of butyrate-producing anaerobes may reduce epithelial oxygen consumption and increase luminal oxygen availability. In a streptomycin-treated Salmonella model, this shift favored facultative anaerobic pathogens (65), but the extent to which the same mechanism operates in human sepsis remains uncertain.

HIF effects remain stage and context dependent. DMOG pretreatment before cecal ligation and puncture increased mucosal HIF-1α and tight-junction proteins and reduced permeability and inflammation, but the design does not establish benefit from treatment after infection (66). Conversely, YC-1 reduced HIF-1α, myosin light-chain kinase, and barrier dysfunction in cytokine-stimulated Caco-2 cells, although off-target effects limit attribution (67). When hemodynamics and gastrointestinal tolerance permit, gradual enteral nutrition may support mucosal substrate supply (68). Fecal microbiota transplantation and short-chain fatty acids have improved barrier or survival outcomes in septic animals (69, 70), while gut-restricted HIF stabilization remains untested in sepsis (71). Because fecal transplantation carries transmission and bacteremia risks (72), intestinal HIF-related metabolism should be judged by feeding tolerance, motility, intestinal fatty acid-binding protein, permeability, mucus, bacterial translocation, and short-chain fatty acids rather than total tissue HIF.

### 4.5 Hepatocyte substrate partitioning, Kupffer cell inflammation, and impaired clearance

The liver regulates systemic substrate supply and clears lactate, ammonia, lipids, drugs, and microbial products while sustaining gluconeogenesis, ureagenesis, bile transport, coagulation factors, and acute-phase proteins. Early catecholamine, glucagon, and glucocorticoid responses promote hepatic glycogenolysis and insulin resistance, contributing to stress hyperglycemia. Because glycogen stores are limited, prolonged infection increasingly relies on gluconeogenesis from lactate, glycerol, and amino acids; depletion of glycogen together with impaired gluconeogenesis or reduced intake may contribute to later hypoglycemia in some patients. The glycemic response may therefore change over time rather than follow a uniformly hyperglycemic pattern. Persistent sinusoidal perfusion heterogeneity, cholestasis, endoplasmic reticulum stress, and mitochondrial injury further impair hepatic synthesis and clearance (73). Increased intestinal permeability further exposes Kupffer cells, sinusoidal endothelium, and hepatocytes to portal microbial products, creating bidirectional gut-liver interactions (74).

Hepatic zonation shapes HIF effects. Oxygen-rich periportal hepatocytes favor gluconeogenesis, fatty acid oxidation, and urea synthesis, whereas relatively hypoxic pericentral cells favor glycolysis, lipogenesis, and drug metabolism (75). Sepsis-associated sinusoidal perfusion heterogeneity may accentuate this pre-existing oxygen gradient (73, 75). As a general HIF response, HIF-1α can reduce mitochondrial oxygen use by promoting glycolysis and pyruvate dehydrogenase kinase expression (7), but sustained glycolysis cannot support all hepatic synthetic and transport functions. Importantly, hepatocyte-specific deletion of HIF-1α, HIF-2α, or both did not substantially alter survival or major circulating substrates in polymicrobial sepsis, despite hepatocyte HIF activation (76), arguing against a single hepatocyte HIF mechanism.

Fatty acid competence appears important. Hepatocyte PPARα deficiency impairs the switch toward lipid use and causes hypoglycemia, defective ketogenesis, steatosis, and lower survival during bacterial infection (77). Reduced HNF4α activity in polymicrobial sepsis is associated with lower PPARα, altered lipid genes, abnormal acute-phase responses, and organ injury (78). HIF-1α and HIF-2α can suppress PGC-1α-related oxidation and promote lipid accumulation in hypoxic or non-septic models, but subtype, zonation, and model differences limit extrapolation (79). When hepatic oxidation declines despite abundant circulating fatty acids, lipid intermediates accumulate and substrate supply becomes mismatched with utilization.

Lactate clearance can decline during hyperdynamic septic shock even when total hepatic blood flow is not markedly reduced, implicating impaired uptake, cofactor regeneration, or gluconeogenesis rather than hypoperfusion alone (80). Blood lactate nevertheless reflects peripheral production, adrenergic stimulation, extrahepatic use, and renal as well as hepatic clearance. Impaired ureagenesis and glutamine metabolism can also raise ammonia and alter astrocyte osmotic balance and neurotransmitter metabolism. Kupffer cell glycolysis supports early antimicrobial activity but can later amplify inflammation; mesenchymal stem cell-derived extracellular vesicles reduced HIF-1α-dependent Kupffer-cell glycolysis and hepatic injury experimentally (81), without proving that systemic HIF inhibition is beneficial. Recovery should therefore be assessed through dynamic clearance and synthetic function—bilirubin, coagulation, glucose, lactate, ammonia, bile acids, triglycerides, and acute-phase proteins—in addition to aminotransferases.

### 4.6 Bioenergetic imbalance, neuroinflammation, and cognitive dysfunction

Sepsis-associated encephalopathy may manifest as delirium, impaired consciousness, or disturbances of attention and memory and may lead to long-term cognitive decline without direct central nervous system infection (82). Neurons require continuous oxidative ATP for membrane potential, synaptic transmission, neurotransmitter cycling, and calcium homeostasis. Endothelial cells, pericytes, astrocytes, microglia, and neurons form a neurovascular unit that coordinates perfusion, barrier transport, and substrate use (83). Blood-brain barrier disruption can permit greater entry of circulating cytokines, damage-associated molecules, microbial products, metabolites, and drugs, thereby altering the metabolic and inflammatory environment of glial cells and neurons. The contribution of this pathway to cognitive dysfunction is likely modified by cerebral autoregulation, regional perfusion, hypoxemia, sedation, and hepatic or renal clearance.

Microglia connect peripheral inflammation with cerebral immunometabolism. Pathogen- and damage-associated signals, cytokines, and hypoxia increase microglial glucose uptake and HIF-1α-related glycolysis. A 2024 study associated acute hyperglycemia with delirium in patients and linked high glucose to ChREBP/HIF-1α signaling, inflammatory glycolysis, barrier injury, and cognitive abnormalities in cecal ligation and puncture rats and BV2 cells (84). Its retrospective and experimental design does not establish direct causality in patients. Other lipopolysaccharide studies show that inhibiting glucose transport or glycolysis reduces microglial inflammatory mediators (85). Acute hyperglycemia may therefore support inflammatory metabolism without restoring neuronal ATP, whereas later hypoglycemia may further restrict neuronal substrate supply. Glycemic trajectory is consequently more informative than a single glucose measurement.

Astrocytes have glycogen reserves and glycolytic capacity and may provide lactate to neurons, whereas neurons rely more heavily on oxidative phosphorylation (86). This metabolic division of labor is derived mainly from basic neuroscience and neurodegeneration studies and may not fully apply across different phases of sepsis. Lactate is useful only when neuronal mitochondria retain oxidative capacity. Experimental sepsis produces persistent brain mitochondrial dysfunction associated with later cognitive impairment; enhancing mitochondrial biogenesis or autophagy improves ATP, oxygen consumption, and behavior (87). Hippocampal HIF-1α/BNIP3L abnormalities have also been associated with mitochondrial injury and cognitive decline, but systemic pharmacological inhibition cannot identify the responsible brain cell type (88).

Cerebral mismatch therefore combines inflammatory glycolysis in microglia with inadequate neuronal oxidative energy for synaptic function. C1q-mediated synaptic elimination is associated with sepsis-related cognitive impairment but lacks evidence of direct HIF-1α control (89). Similarly, short-chain fatty acid/GPR43 signaling can influence cognition indirectly without establishing a brain HIF mechanism (90). Studies should distinguish these modifiers from direct HIF effects and combine cell localization with barrier integrity, microglial glycolysis, neuronal respiration, synaptic or network function, delirium and cognitive assessments, cerebral perfusion or oxygenation, and circulating glucose, lactate, ammonia, drug exposure, and organ clearance. The cell-specific HIF-1α-associated metabolic responses and their organ-level functional consequences are summarized in **Table 1**.

**Table 1:** Cell-resolved HIF-1α-associated metabolic responses and functional consequences across organs in sepsis.

Organ	Cell type/source	Key metabolic pathway	Adaptive/early role	Persistent effect and functional consequence	References
Lung	Alveolar macrophage	HIF-1α–PKM2 glycolysis	Antimicrobial defense	Inflammation; barrier injury	(8, 9, 11, 13)
	Neutrophil	HIF-supported glycolysis	Migration; pathogen killing	NETs/ROS; tissue injury	(35)
	Pulmonary endothelial cell	GLUT1/glycolysis; HIF-FOXM1	Survival; vascular repair	Hyperpermeability	(36–39)
	AT2 epithelial cell	Glycolysis; FAO; mitophagy	Survival; epithelial repair	Lipid imbalance; repair failure	(40–42)
Kidney	Proximal tubular cell	Glycolysis; FAO; NAD+–PGC-1α	Energy saving; mitophagy	ATP↓; transport/repair failure	(28, 45–52)
	Renal macrophage	NF-κB–HIF glycolysis	Innate defense	Tubular inflammation	(45)
Heart	Cardiomyocyte	HIF–PDK4/PDH; FAO/OXPHOS	Substrate flexibility	ATP↓; contractile dysfunction	(26, 53–60)
Intestine	Enterocyte/colonocyte	Butyrate oxidation–HIF; glycolysis	Barrier maintenance	Permeability↑; translocation	(62–67)
	Gut microbiota	SCFA production	Butyrate supply	Dysbiosis; SCFAs↓	(65, 69–72)
Liver	Hepatocyte	PPARα/HNF4α–FAO; HIF	Substrate handling	Clearance↓; cholestasis	(75–80)
	Kupffer cell	HIF-1α–dependent glycolysis	Phagocytosis	Inflammation; liver injury	(73, 74, 81)
	LSEC	Perfusion; substrate exchange	Microvascular exchange	Oxygen delivery↓	(73, 74)
Brain	Microglia	HIF-related glycolysis	Innate response	Neuroinflammation; BBB injury	(84, 85)
	Astrocyte	Glycolysis; lactate support	Neuronal support	Neurovascular uncoupling	(83, 86)
	Neuron	OXPHOS; glucose/lactate oxidation	Synaptic ATP supply	Synaptic/cognitive dysfunction	(82, 86–88)
	Brain endothelium/pericyte	BBB transport; perfusion	BBB integrity	Hypoperfusion; barrier failure	(82, 83)

## 5 Metabolic interventions and clinical translation

### 5.1 Targeted modulation of HIF-1α and related metabolic pathways

HIF-targeted strategies may have different consequences according to cell identity, organ energy demand, infection control, signal intensity, and disease phase. Short-term stabilization may support hypoxic tolerance in renal tubular, intestinal epithelial, and other barrier cells (40, 45, 47, 66), whereas persistence in pulmonary, hepatic, or cerebral myeloid cells can prolong inflammatory glycolysis (34, 81, 84, 85). Excess cardiomyocyte HIF-1α has been linked more directly to impaired respiration and contraction (57). Conversely, systemic inhibition could compromise epithelial or endothelial survival and repair. A favorable pathway change would therefore be more convincing when accompanied by improved organ function without impaired pathogen control.

Patient heterogeneity further complicates selection. Similar lactate levels may reflect hypoperfusion, catecholamine-driven glycolysis, restricted mitochondrial oxidation, or impaired hepatic and renal clearance. A 2025 analysis of serial serum samples from two septic shock trial cohorts identified three metabolic subphenotypes; a persistently low-lysophospholipid profile was associated with poor outcome, and some patients changed class over time (91). This supports longitudinal phenotyping but does not yet guide selection of HIF-, glycolysis-, or mitochondria-directed drugs. Potential variables include hemodynamics, vasoactive dose, glucose, lactate and the lactate-to-pyruvate ratio, ketones, free fatty acids, acylcarnitines, acid-base status, and liver and kidney function. Stable isotope tracing can distinguish plasma concentration from flux in mechanistic and small clinical studies.

HIF prolyl hydroxylase inhibitors stabilize HIF-1α and HIF-2α and are used clinically mainly for anemia in chronic kidney disease (92); clinical benefit in sepsis-associated organ injury has not been established. Because HIF effects vary across cells and disease phases, their evaluation in sepsis may need to consider timing, tissue exposure, pathogen burden, and organ function. Gut-restricted inhibitors and myeloid-targeted nanocarriers may offer greater selectivity but remain preclinical (71, 93).

Modulation of glycolytic enzymes may alter inflammatory metabolism without directly changing HIF stability. For PKM2, it is useful to distinguish cytosolic catalytic activity from nuclear transcriptional regulation. DASA-58 and TEPP-46 promote active tetramers, reduce nuclear PKM2–HIF-1α cooperation, and suppress interleukin-1β in lipopolysaccharide-stimulated macrophages (9). PKM2 knockdown or shikonin reduces lactate and high-mobility group box 1 and improves outcomes in experimental endotoxemia or polymicrobial sepsis (94), but global inhibition is metabolically distinct from tetramer stabilization, and shikonin has additional cytotoxic and redox effects. Systemic inhibition of lactate dehydrogenase A or PFKFB3 could also reduce short-term energy supply in barrier and hypoxic parenchymal cells. Cell-selective delivery, limited exposure, and pathogen monitoring may help evaluate these potential trade-offs in preclinical studies.

Pyruvate dehydrogenase kinase inhibition may partially reconnect glycolysis with mitochondrial glucose oxidation. PDK4 inhibition improves myocardial pyruvate oxidation, contraction, and mitochondrial structure in septic animals (60). Dichloroacetate activates the pyruvate dehydrogenase complex across several PDK isoforms and has improved hepatic oxidation, systemic energy balance, bacterial clearance, and survival in cecal ligation and puncture mice (95–97). Whether these effects translate to human organ recovery remains uncertain. Increasing pyruvate entry may provide limited benefit when respiratory reserve is markedly reduced and could increase reducing equivalents or reactive oxygen species. Assessment may therefore include pyruvate dehydrogenase activity, substrate flux, oxygen consumption, respiratory reserve, redox balance, pathogen burden, and organ function.

Mitochondrial quality control is also phase- and context-dependent. Early mitophagy may remove depolarized mitochondria, whereas recovery is likely to require coordinated mitochondrial biogenesis, NAD+ regeneration, and antioxidant capacity (13, 29, 42, 47, 52). Persistent clearance without adequate replacement may reduce mitochondrial mass, while excessive reactive oxygen species scavenging could weaken antimicrobial signaling. Autophagic flux, mitochondrial abundance, membrane potential, and respiratory reserve provide more information than single measurements of LC3, PINK1, or PGC-1α. Organ priorities also differ: inflammatory glycolysis and barrier repair in the lung; pyruvate and fatty acid oxidation in the heart; hypoxic tolerance followed by tubular redifferentiation in the kidney; epithelial HIF–short-chain fatty acid coupling in the intestine; metabolic clearance in the liver; and microglial inflammation versus neuronal oxidation in the brain. A pathway change would be more clinically relevant when it is accompanied by improvement in the corresponding organ function.

Candidate interventions may be more informative when matched to a biologically defined state rather than to elapsed time alone. Uncontrolled infection, high myeloid glycolysis, preserved or exhausted mitochondrial reserve, and the dominant organ phenotype could lead to different treatment priorities within the same day of illness. Serial sampling may be useful because patients can move between metabolic subphenotypes (91). Lactate-to-pyruvate ratios, acylcarnitines, and circulating lipids are potentially informative only when sampling conditions, adrenergic therapy, nutrition, and organ clearance are considered. At present, these measures are better suited to mechanistic stratification and trial enrichment than to routine selection of a particular HIF- or metabolism-directed drug.

Translation would also benefit from explicit assessment of safety and distribution. HIF prolyl hydroxylase inhibition may alter erythropoiesis, iron handling, vascular growth, and coagulation, while glycolytic inhibition may impair leukocyte pathogen killing or epithelial survival. Nanocarriers can redistribute toward the liver and spleen through protein corona formation and mononuclear phagocyte uptake, and septic perfusion or organ failure may further change exposure (92, 93). Translational studies could combine pharmacokinetics with cell-specific target engagement, pathogen burden, antimicrobial treatment, and organ endpoints. Dose, duration, and reversibility remain important because partial transient modulation may differ substantially from genetic deletion or continuous pathway blockade.

### 5.2 Indirect modulation by nutrition and microbiota-targeted strategies

Nutrition changes the availability of glucose, lipids, and amino acids but does not guarantee cellular ATP production. Critical care guidelines support gradual enteral nutrition after relative hemodynamic stabilization and caution when vasopressor requirements rise or intestinal ischemia is suspected (98, 99). NUTRIREA-2 found no mortality or infection advantage of early isocaloric enteral over parenteral nutrition in ventilated patients with shock and reported more gastrointestinal complications with enteral feeding (100). NUTRIREA-3 found that early low-calorie, low-protein feeding accelerated recovery and reduced some complications compared with standard targets, without a mortality difference (101). These trials support caution against rapid full feeding during shock but do not establish an HIF-mediated benefit.

Nutritional interventions are relevant to this review because they reshape the substrate environment. When the respiratory chain, cofactor regeneration, or fatty acid oxidation is restricted, additional glucose or lipid may accumulate as lactate, lipid intermediates, or acylcarnitines instead of increasing ATP. During recovery, adequate energy and protein remain necessary for mitochondrial renewal, tissue repair, and rehabilitation. Feeding intensity should therefore reflect perfusion, gastrointestinal tolerance, clearance capacity, and changing workload. Indirect calorimetry and isotope tracing can investigate substrate use, but nutritional targets should not be dictated by HIF-1α, glucose, or lactate alone (102).

Fermentable substrates and microbial metabolites also influence epithelial HIF indirectly. Fecal microbiota transplantation or short-chain fatty acids have improved barrier or survival outcomes in septic animals (69, 70), but clinical efficacy and timing are unknown. The PROSPECT trial found that Lactobacillus rhamnosus GG did not reduce ventilator-associated pneumonia and caused rare strain-related infections (103). Microbiota-directed interventions should therefore be regarded as indirect support of the substrate environment and barrier, not established HIF-targeted therapy. The principal context-matched metabolic interventions and their translational limitations are summarized in **Table 2**.

**Table 2:** Context-matched metabolic interventions and translational considerations in sepsis.

Strategy/approach	Target/phase	Intended effect	Major caveat	References
HIF modulation: PHD or HIF inhibition	Cell- and phase-selected	Hypoxia tolerance or inflammatory control	Opposing organ effects; HIF-2α/EPO/iron	(40, 45, 47, 57, 66, 71, 92)
Cell-selective delivery	Myeloid cells or gut	Local effect; systemic exposure↓	Distribution; liver/spleen uptake	(71, 93)
PKM2/glycolysis modulation	Persistent myeloid activation	Inflammatory glycolysis↓	Antimicrobial/barrier glycolysis↓	(9, 94)
PDK inhibition: PDK4/DCA	Heart/liver; respiration preserved	PDH and pyruvate oxidation↑	Limited effect with ETC failure	(60, 95–97)
Mitochondrial restoration	Recovery; high-energy cells	NAD+; biogenesis; reserve↑	Mitophagy–biogenesis imbalance	(13, 29, 42, 47, 52, 87)
Gradual enteral nutrition	Hemodynamic stability	Match supply to utilization	Overfeeding; intolerance	(98–102)
Microbiota/SCFA support	Gut barrier dysfunction	Butyrate; barrier support	Limited evidence; infection risk	(69, 70, 103)

## 6 Discussion

### 6.1 Metabolic flexibility and organ-specific metabolic mismatch

HIF-1α effects are determined by the expressing cell and its functional workload. Pulmonary myeloid glycolysis may prolong inflammation, while epithelial and endothelial HIF signaling supports repair (34, 39, 40). Renal tubular hypoxic adaptation cannot replace recovery of fatty acid oxidation and mitochondrial biogenesis (45, 52). Sepsis impairs myocardial pyruvate oxidation and contractile function (55, 60), whereas cardiomyocyte-specific evidence directly implicates excessive HIF-1α in mitochondrial respiratory and contractile dysfunction (57). Intestinal short-chain fatty acid–oxygen–HIF coupling supports the barrier (62, 66), and cerebral microglial glycolysis must be distinguished from neuronal oxidative insufficiency (84, 87, 88). Total tissue HIF-1α is therefore an aggregate signal that cannot specify a cell’s metabolic state or predict organ outcome.

Metabolites and damage signals also connect cell populations and organs. Lactate, succinate, reactive oxygen species, and cytokines from immune cells alter endothelial and parenchymal HIF and substrate use, while mitochondrial components and oxidized lipids from injured parenchyma feed back on immune recruitment (8, 13, 20, 21). Hepatic and renal clearance, intestinal barrier integrity, and microvascular perfusion reshape the substrate environment of distant organs (73, 74). Cell-resolved localization, flux, and organ function are consequently more informative than tissue HIF expression alone.

Metabolic flexibility is preserved when early glycolysis is followed by recovery of pyruvate and fatty acid oxidation, mitochondrial biogenesis, and differentiation. Persistent inefficient energy production, lipid intermediate accumulation, depleted respiratory reserve, and dedifferentiation indicate organ-specific metabolic mismatch (13, 29, 52). The threshold differs by cell: intestinal epithelium and some myeloid cells tolerate temporary glycolysis better than proximal tubules, cardiomyocytes, or neurons. Neither HIF-1α elevation nor glycolysis alone distinguishes adaptation from dysfunction. For experimental or clinical assessment, mismatch would be more plausible when preserved or increased substrate availability or glycolysis coexists with restricted pyruvate or fatty acid oxidation, reduced respiratory reserve, metabolite accumulation, and delayed recovery of an organ-specific function.

The mismatch framework does not imply that reduced oxidative metabolism is intrinsically harmful. Lowering transport, synthesis, or contractile activity can be an energy-conserving response when oxygen use is temporarily constrained. The decisive issue is whether this suppression remains proportionate to infection control and is reversible as cellular conditions improve. A compatible trajectory would include preservation of viability, removal of damaged mitochondria, renewed respiratory capacity, and recovery of organ-specific work. An incompatible trajectory would combine persistent low ATP yield with declining respiratory reserve, lipid or reducing-equivalent accumulation, dedifferentiation, and incomplete functional recovery. This distinction explains why identical changes in HIF-1α, lactate, or glycolytic enzymes can carry different meanings across cells and time and why molecular normalization is neither necessary nor sufficient evidence of organ recovery. This also distinguishes mismatch from isolated mitochondrial dysfunction and from adaptive hypometabolism, which may remain reversible and proportionate to a temporary reduction in organ workload.

### 6.2 Disease phase, immunometabolic heterogeneity, and interorgan coupling

Early HIF-related glycolysis supports antimicrobial myeloid activity and hypoxic tolerance in selected barrier and tubular cells (11, 34, 35, 39, 40, 45, 66). Persistent pathogens, succinate, oxidative stress, and mitochondrial damage maintain inflammation during progression (8, 13). Recovery shifts the requirement toward pyruvate and fatty acid oxidation, mitochondrial biogenesis, and redifferentiation in energy-demanding cells (29, 52, 55, 60). Identical pathway modulation may therefore have opposite effects at different times, making serial experimental and clinical assessment essential.

Progressive sepsis is not uniformly hyperglycolytic. Some monocytes and lymphocytes suppress both glycolysis and oxidative phosphorylation as immune function declines (10). Single-cell studies show concurrent inflammatory, low-antigen-presentation, and immunosuppressive states, with abnormal neutrophils and emergency granulopoiesis associated with immunosuppressive endotypes (104, 105). Active glycolysis in local myeloid cells can thus coexist with peripheral immune hyporesponsiveness in the same patient, which circulating lactate or bulk glycolytic markers cannot resolve.

This coexistence argues against assigning one metabolic stage to an entire patient. A lung macrophage population may remain glycolytically inflammatory while circulating monocytes are tolerant and renal tubules are attempting oxidative recovery. Endothelial dysfunction can simultaneously restrict local oxygen delivery and alter drug and metabolite exchange. The clinically relevant unit may therefore be a cell-, organ-, and time-specific metabolic state linked to a defined function. Serial, compartment-aware sampling is difficult in humans, but blood endotypes can be integrated with organ imaging, clearance tests, physiological endpoints, and tissue- or urine-derived markers to reduce reliance on a single systemic classification.

Interorgan coupling further complicates interpretation. Lactate can be cleared by the liver or oxidized by the heart; renal failure impairs organic acid, nitrogen metabolite, and drug excretion; hepatic dysfunction reduces lactate, ammonia, lipid, and drug metabolism; and barrier failure increases portal and systemic exposure to microbial products (21, 22, 43, 61, 73, 74). Circulating cytokines and mitochondrial damage-associated molecular patterns transmit systemic stress signals (13), while endothelium regulates oxygen delivery, tissue drug exposure, and metabolite exchange (18, 38, 39). Reversible hypometabolism and persistent mismatch probably form a continuum and should be distinguished through longitudinal metabolic assessment, mitochondrial function, and organ outcomes rather than attributed to a single HIF pathway.

### 6.3 Static biomarkers and dynamic metabolic phenotyping

Expression of HIF-1α, glucose transporter 1, lactate dehydrogenase A, or pyruvate dehydrogenase kinase indicates metabolic potential, not actual flux or ATP efficiency. Stable isotope tracing measures substrate turnover and carbon fate; targeted or untargeted metabolomics measures composition; and extracellular flux analysis and high-resolution respirometry assess glycolysis and mitochondrial respiration (102). These methods should be integrated because plasma concentrations are shaped by multiple organs and do not map directly to cell-specific metabolism.

Single-cell and spatial transcriptomic, epigenomic, and metabolomic approaches reveal cell heterogeneity obscured by tissue averages. However, pathway enrichment remains an expression-level tendency and requires validation by localization, tracing, respiration, and organ function (104, 105). Longitudinal sampling is equally important because pathogen control, short-term functional improvement, oxidative recovery, and structural repair are asynchronous.

Clinical phenotyping may combine lactate trajectories, glycemic variability, ketones, free fatty acids, acylcarnitines, amino acid profiles, clearance capacity, mitochondrial injury, and organ function. Metabolomic and transcriptomic studies identify changing septic shock subphenotypes and immune endotypes (91, 106), but their stability and therapeutic relevance remain unvalidated. Serial changes and responses to intervention may better reflect metabolic reserve than a single baseline value.

A practical dynamic panel should pair molecular and functional levels. HIF nuclear localization and enzyme expression can define pathway engagement; isotope tracing or extracellular flux can estimate substrate use; respirometry can quantify oxidative capacity and reserve; and organ-specific measures can determine whether the metabolic change is accompanied by functional recovery. For example, improved renal respiration should coincide with transport or filtration recovery, and altered cardiac substrate oxidation should coincide with load-adjusted contractile improvement. Repeated measurements before and after intervention may reveal metabolic reserve more reliably than a baseline concentration and can help distinguish recovery, pharmacological target engagement, and nonspecific changes caused by nutrition or clearance. Such composite assessment remains exploratory and requires prospective validation before clinical thresholds can be defined.

### 6.4 Key controversies and evidence limitations

Apparently conflicting HIF findings often arise from different cell types, parallel pathways, and observation times. Myeloid HIF supports glycolytic adaptation, cell survival, and context-dependent antimicrobial and inflammatory functions (11, 34, 35); barrier and tubular HIF can support short-term adaptation (39, 40, 45); and excessive cardiomyocyte HIF impairs respiration (57). NRF2/HO-1, ferroptosis, and mitophagy intersect with HIF but are not components of a single downstream pathway. Tubular HIF-1α/BNIP3 evidence is largely derived from ischemia–reperfusion models (47), whereas NRF2 and ferroptosis evidence derives mainly from experimental sepsis-associated kidney injury (48, 50, 51). HIF-related mitophagy is most directly supported by BNIP3/BNIP3L evidence in selected renal and cerebral models, whereas PINK1/Parkin and other stress pathways may act independently. Controlled mitochondrial clearance may limit ROS and damage signals, but persistent clearance without adequate biogenesis may reduce respiratory reserve. Ferroptosis is connected to HIF mainly through intersecting iron, lipid-peroxidation, and redox networks; current sepsis evidence is strongest in renal tubular injury rather than as a universal direct HIF-1α pathway (42, 47–51, 88).

Model differences limit comparison. Lipopolysaccharide reproduces acute inflammatory signaling but not persistent infection, clearance, or antimicrobial treatment. Cecal ligation and puncture includes polymicrobial infection and tissue injury, but severity depends on surgery, resuscitation, and antimicrobial protocols. Clinical sepsis adds diverse pathogens, sources, treatments, and organ phenotypes. The Minimum Quality Threshold in Preclinical Sepsis Studies emphasizes infection type, resuscitation, antimicrobials, organ function, and humane endpoints (107). Ventilation, vasoactive drugs, anesthesia, temperature, and nutrition should also be reported because they alter oxygen and substrate flux.

No single biomarker confirms metabolic recovery. Stronger assessment combines substrate flux, ATP, redox state, mitochondrial abundance, membrane potential, respiratory reserve, autophagic flux, and cell-specific functions. Animal sex, age, feeding, temperature, anesthesia, and resuscitation must be controlled and reported according to ARRIVE 2.0 principles (108). *In vitro*, high-dose lipopolysaccharide in isolated parenchymal cells cannot reproduce immune infiltration, microcirculation, substrate competition, or interorgan clearance. HK-2 cells retain some proximal tubular features but incompletely model transporter expression and metabolic reserve (109, 110). Primary cells, organoids, co-culture, and cell-specific interventions are needed for causal validation.

Human evidence remains dominated by plasma metabolites, circulating immune cells, and blood omics; direct organ- and cell-specific flux data are scarce. Definitions of sepsis phase, metabolic recovery, and organ dysfunction also vary. Metabolic flexibility and organ-specific metabolic mismatch therefore remain comparative mechanistic concepts rather than validated clinical classifications. Operational definitions, reproducible measurement panels, and thresholds are required before they can predict organ recovery or treatment response. The evidence levels and criteria for HIF-1α causal attribution are summarized in **Table 3**.

**Table 3:** Evidence hierarchy for interpreting HIF-1α-related metabolic findings in sepsis.

Evidence/model	Representative source or readout	Minimum basis for HIF attribution	Interpretation and key limitation	References
**Human organ-specific**	Tissue; arteriovenous balance; PET/MRS; isotope tracing	Cell-localized HIF plus organ flux or function	Organ association or net flux; rare, invasive, and low cell resolution	(18, 19, 82)
**Human systemic**	Plasma metabolomics; lactate; PBMC omics	HIF activity in sampled blood cells only	Systemic phenotype only; production, utilization, and clearance are conflated	(11, 91, 104–106)
**Animal sepsis**	CLP, cecal slurry, or live infection; conditional knockout	Cell-specific Hif1a perturbation plus flux and function	Model-level causality; species, severity, treatment, and timing dependent	(26, 57, 66, 76)
**LPS/endotoxemia**	*In vivo* endotoxemia; LPS-stimulated cells	HIF perturbation plus target engagement	Acute inflammatory mechanism; lacks persistent infection and clearance	(8, 9, 34, 94)
***In vitro* or organotypic**	Primary cells, cell lines, co-culture, or organoids	HIF gain/loss or HRE evidence plus flux and function	Cell-autonomous mechanism; lacks perfusion and interorgan workload	(9, 45, 67, 84)
**Non-sepsis mechanistic**	Hypoxia, ischemia–reperfusion, colitis, or fibrosis	Pathway plausibility unless replicated in sepsis	Mechanistic hypothesis only; indirect and disease-phase uncertain	(47, 64, 71, 79)

Bold text in the first column identifies model categories.

Cross-cohort comparison also requires harmonized sampling times, organ dysfunction definitions, supportive-treatment data, and preanalytical handling. Without these controls, differences in nutrition, catecholamine exposure, clearance, and sample processing may be mistaken for biological metabolic subtypes.

## 7 Conclusions and future perspectives

HIF-1α connects hypoxia sensing, inflammation, substrate utilization, and mitochondrial function, but its direction of effect depends on cell identity, organ energy demand, infection control, and disease stage. Early glycolysis can support antimicrobial activity and transient hypoxic tolerance. When sustained with restricted pyruvate and fatty acid oxidation and failed mitochondrial renewal, it reduces metabolic flexibility and disconnects energy production from organ maintenance and repair.

Organ differences mean that global HIF activation or inhibition is unlikely to provide uniform benefit. Tissue HIF-1α, lactate, or a single metabolic protein cannot distinguish adaptation from persistent dysfunction. NRF2/HO-1, ferroptosis, C1q-mediated synaptic elimination, and GPR43 should be treated as intersecting, parallel, or indirect mechanisms according to the evidence. Therapeutic studies should align HIF-, glycolysis-, pyruvate-oxidation-, mitochondrial-, nutritional-, or microbiota-directed interventions with the target cell, organ, infection status, and treatment window, while evaluating organ function rather than molecular markers alone.

Future work should establish operational definitions of metabolic flexibility and organ-specific metabolic mismatch through longitudinal sampling. Single-cell and spatial omics should be integrated with stable isotope tracing, metabolic flux, high-resolution respirometry, and organ endpoints to determine causality across cells and phases and to characterize interorgan substrate competition. More clinically representative models and prospective metabolic phenotyping are also required. Translation would be supported more convincingly when an intervention preserves pathogen clearance while improving organ recovery and long-term outcomes. Because direct human organ-specific metabolic flux data remain limited, these approaches should currently be regarded as research strategies rather than established tools for treatment selection.

An operational definition could combine the magnitude and reversibility of a stress-induced substrate shift with recovery of respiratory reserve and the relevant organ function. Prospective studies should test whether such composite trajectories identify reproducible patient subgroups, predict delayed organ recovery, or modify treatment response. Experimental platforms should incorporate infection control, resuscitation, antimicrobial exposure, nutrition, age, and comorbidity so that metabolic interventions are evaluated in a clinically realistic context. The central translational criterion is restoration of cell- and phase-appropriate organ function without compromising antimicrobial defense or causing injury in another organ.
